# *Limosilactobacillus reuteri* DSM 17938-Containing Infant Formulas and the Associations with Gastrointestinal Tolerance: A Cross-Sectional Observational Study

**DOI:** 10.3390/nu15030530

**Published:** 2023-01-19

**Authors:** Happy Tummy Consortium, Luca Lavalle, Nicolas Sauvageot, Colin Ivano Cercamondi, Ivana Jankovic, Delphine Egli, Yvan Vandenplas

**Affiliations:** 1 Biostatistics & Data, Nestlé Research, 1000 Lausanne, Switzerland; 2 Nestlé Product Technology Center—Nutrition, Société des Produits Nestlé S.A., 1800 Vevey, Switzerland; 3 UZ Brussel, KidZ Health Castle, Vrije Universiteit Brussel (VUB), 1090 Brussels, Belgium

**Keywords:** infant, nutrition, probiotics, gastrointestinal tolerance, colic, infant formula

## Abstract

*Limosilactobacillus* (*L*.; previously *Lactobacillus*) *reuteri* has been shown to influence gastrointestinal (GI) tolerance. This study was a secondary analysis of GI tolerance data from a multi-country, cross-sectional, observational study in healthy infants using the validated Infant Gastrointestinal Symptom Questionnaire (IGSQ) and a gut comfort questionnaire. Breastfed infants (BFI; *n* = 760) were compared to formula-fed infants receiving either *L. reuteri*-containing formula (FFI + LR; *n* = 470) or standard formula without any probiotic or prebiotic (FFI-Std; *n* = 501). The IGSQ composite scores (adjusted mean ± SE) in FFI + LR (22.17 ± 0.39) was significantly lower than in FFI-Std (23.41 ± 0.37) and similar to BFI (22.34 ± 0.30;), indicating better GI tolerance in FFI + LR than in FFI-Std. Compared with FFI-Std, FFI + LR had lower reports of difficulty in passing stools (11% vs. 22%; adjusted-odds ratio (OR) (95%CI) = 0.46 (0.31–0.68)), fewer hard stools (mean difference = −0.12 (−0.21, −0.02)) and less physician-confirmed colic (OR = 0.61 (0.45–0.82)), and similar to BFI. Parent-reported crying time (mean difference = −0.15 (−0.28, −0.01)), frequency of spitting-up/vomiting (mean difference = −0.18 (−0.34, −0.03)), volume of spit-up (mean difference = −0.20 (−0.32, −0.08)) and fussiness due to spitting-up/vomiting (mean difference = −0.17 (−0.29, −0.05)) were lower in FFI + LR versus FFI-Std and similar to BFI. In this study, *L. reuteri*-containing formula was associated with improved digestive tolerance and behavioral patterns.

## 1. Introduction

Healthy term infants who are formula-fed often show signs of feeding intolerance in the first months of life [1,2] which may manifest in gastrointestinal (GI) symptoms such as infrequent or hard stools, spitting-up, or flatulence, or behaviorally as fussiness, crying, or dysregulated sleep. The underlying etiology of these GI symptoms and behaviors is thought to be multifactorial but has yet to be fully elucidated. Infants who are breastfed often have less GI intolerance compared with those who are formula-fed. For example, formula-fed infants generally have firmer, less frequent stools compared to breastfed infants [1,2], likely due to differences in the lipid and mineral fractions of the stools [3] and the presence of bioactive molecules in human milk [4]. In addition, undesirable GI effects [1,3,5] such as colic, flatulence and regurgitation, while relatively common in infants overall [5,6], are less common in breastfed infants [7,8]. Parental concern around GI effects is high with a large proportion of parents switching formulas for reasons such as regurgitation or vomiting or restless behavior [9,10]. Thus, while breastfeeding is the gold standard of infant nutrition, for infants who are formula-fed, it is important that stooling patterns, GI tolerance and associated behaviors are comparable to breastfed infants.

In randomized controlled trials (RCTs), addition of probiotics has been shown to be effective in improving GI tolerance in infants [11]. In particular, *Limosilactobacillus* (*L*.; previously categorized as *Lactobacillus*) *reuteri* DSM 17938, was reported to be effective in the prevention of colic and regurgitation, particularly in breastfed infants [12]. However, effects demonstrated in a RCT can sometimes translate differently outside of a controlled trial setting, particularly for patient/parent-reported outcomes [13]. Therefore, understanding the effectiveness of *L. reuteri*-containing formula in real-world settings is important as it provides complementary evidence to the findings from controlled trial settings, but such data are not currently available. In a large cross-sectional observational study, GI tolerance of infants who received formula containing any prebiotics or probiotics or a combination was non-inferior to breastfed infants [14]. In addition, these infants had better GI outcomes than infants who had received formula without the addition of any prebiotics or probiotics. In order to evaluate the effectiveness of *L. reuteri* alone, as part of an infant formula matrix, we thus conducted a secondary analysis and compared GI-tolerance outcomes among three sub-populations including: (1) exclusively or predominantly breastfed infants (BFI), (2) infants receiving *L. reuteri* (DSM 17938)-containing formulas (FFI + LR), or (3) infants consuming standard formulas without any probiotic or prebiotic (FFI-Std).

## 2. Materials and Methods

### 2.1. Design and Participants

The data for the current analysis came from a multi-center, cross-sectional, observational study conducted in six countries (Egypt, Pakistan, Philippines, Indonesia, Malaysia and India). Infants aged 6 to 16 weeks were recruited during routine visits and, upon informed consent, study physicians administered two questionnaires to the mothers: Infant Gastrointestinal Symptom Questionnaire (IGSQ) and a feeding practice and gut comfort questionnaire (FPGCQ). Apparently healthy infants (i.e., without acute ongoing, recent, or chronic illness necessitating medical follow up and without food allergies) born full-term to parents aged 18 years or older were eligible. Participating infants were required to be exclusively or predominantly breastfed or formula-fed using the same brand of formula for two weeks or more at the time of recruitment. Exclusive/predominant feeding regimen was defined based on the current regimen: if 75% or more of the daily feeds came from breastmilk, infants were assigned to the breastfed group; if 75% or more of the daily feeds came from a single formula, infants were assigned to the formula-fed group. Study subjects and their parents or the public were not involved in the design, conduct, reporting or dissemination plans of the study.

### 2.2. Outcome Measures

Infant and parent demographics including age at enrollment, sex, gestational age, delivery type, mother’s education level and history of gastrointestinal disease in parents were recorded. Infant weight, height and head circumference at birth were recorded and the three anthropometric parameters were also measured at the time when the study physicians administered the IGSQ and FPGCQ to the parents. The IGSQ composite score (range 13–65), calculated by summing 13 individual item responses, was used to assess overall GI tolerance [15]. Composite scores less than 23 generally indicate no GI distress while scores of 23–30 indicate certain GI distress and scores above 30 indicate clinically meaningful GI distress [15]. Individual IGSQ item scores were used to assess GI symptoms (lower score means less symptoms). The FPGCQ was designed for this study and was used to collect feeding practice information (including counts of breast and formula feeds per day, formula brand where applicable and consumption of solid foods), information on colic and 24-h stooling pattern. Information on physician-confirmed infant colic was obtained using two questions: “Did the child have colic in the past week?” and “Was your child ever diagnosed with colic?”. If the answer to the second question was yes, follow-up questions for age and feeding regimen at time of diagnosis were asked. Colic was defined according to the ROME IV diagnosis criteria: A) an infant who is <5 months of age when the symptoms start and stop; B) recurrent and prolonged periods of infant crying, fussing, or irritability reported by caregivers that occur without obvious cause and cannot be prevented or resolved by caregivers and C) no evidence of infant failure to thrive, fever, or illness. Study investigators explained the colic definition to the mothers when asking the questions. Stooling pattern in the past 24 h included stool frequency and consistency and whether each stool was difficult to pass. Stool consistency was rated on the 4-point Brussels Infants and Toddlers Stool Scale (1-watery, 2-loose, 3-formed, 4-hard), which is validated for non-toilet trained children [16]. Formula brand as reported by the parents or caregivers was used to group formula-fed infants into FFI + LR (infants receiving formula containing *L. reuteri* DSM 17938) and FFI-Std (infants receiving standard formulas without any probiotic or prebiotic).

### 2.3. Statistical Analysis

Descriptive data were summarized using appropriate statistics for continuous and categorical measures. The IGSQ composite score as well as individual IGSQ item scores were analyzed using analysis of covariance (ANCOVA) adjusting for feeding group, study site, infant age, sex, delivery type, history of GI disease in parents and mother’s education. Stooling was compared between feeding groups using logistic regression for difficulty passing stool, a negative binomial model for stool frequency and an ANCOVA model for stool consistency with adjustment for the same covariates as in the IGSQ models. Physician-confirmed colic was modeled using logistic regression with adjustment for the same covariates. All tests were two-sided with a significance level of alpha = 0.05. Analyses were conducted using SAS/STAT software version 9.3 or higher (SAS Institute Inc., Cary, NC, USA). This current analysis reports the secondary analysis of data from a cross-sectional observational study (ClinicalTrials.gov: NCT03703583) [14]; hence, no sample size calculation is available.

### 2.4. Ethics Approval

This study was conducted in accordance with the World Medical Association Declaration of Helsinki. The study was approved by the Institutional Review Boards listed in the “Institutional Review Board Statement” below. Parents or legally authorized representatives of the infants in this study provided written informed consent prior to enrollment.

## 3. Results

### 3.1. Infants’ Characteristics

Data from 470 FFI + LR and 501 FFI-Std as well as 760 BFI were used in this secondary data analysis. Demographic characteristics for the three groups including infant sex, gestational age at birth, age at enrollment, delivery type, history of GI disease and mother’s education are shown in Table 1. The proportion of infants delivered by Caesarean section was higher in FFI-Std and maternal education was lower in FFI + LR (both *p* < 0.01). Weight, height (both *p* < 0.01) and head circumference (*p* < 0.05) at visit were also lower in FFI + LR. Weight, height and head circumference at birth (all *p* < 0.01) were lower in FFI-Std. Differences in anthropometric parameters were deemed to be of minimal clinical importance by the study physicians.

### 3.2. Infant Gastrointestinal Symptom Questionnaire Composite Scores

**Figure 1:**
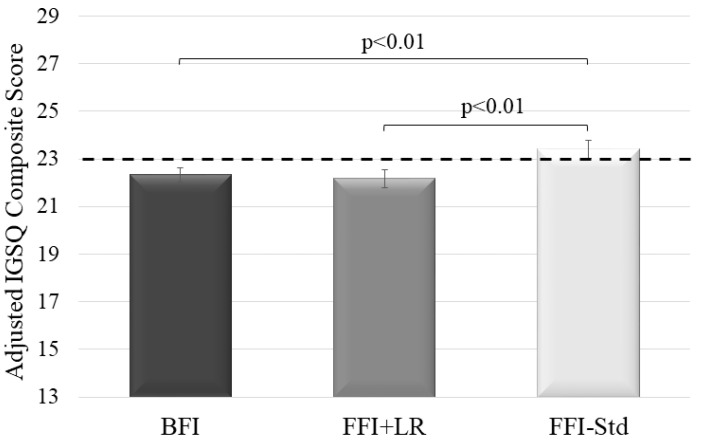
Adjusted mean IGSQ composite scores ± SE by feeding groups. Group comparison done by analysis of covariance adjusted for feeding group, study site, infant age, sex, delivery type, history of GI disease in parents and mother’s education. IGSQ composite score can range from 13–65, with higher values indicating greater discomfort. Dotted line marks threshold of 23 indicating certain GI discomfort (>23 to 30; >30 to 65 indicates GI distress) and essentially no GI issues (≤23). BFI—Breastfed infants; FFI + LR—Infants fed formula with *L. reuteri*; FFI-Std—Infants fed standard formula without any probiotic or prebiotic; GI, gastrointestinal; IGSQ, Infant Gastrointestinal Symptom Questionnaire. *n* = 760 in BFI; *n* = 501 in FFI-Std; *n* = 470 in FFI + LR.

The adjusted mean IGSQ composite score ± SE in FFI + LR (22.17 ± 0.39) was lower than in FFI-Std (23.41 ± 0.37; mean difference: −1.24, 95% CI: −2.19, −0.30; *p* < 0.01) indicating better GI tolerance in FFI + LR than in FFI-Std. BFI (22.34 ± 0.30) also had lower IGSQ composite score than FFI-Std (mean difference: −1.07 (95% CI −1.87, −0.28; *p* < 0.01). The IGSQ composite scores in FFI + LR and BFI were similar (mean difference −0.17; 95% CI −0.98, 0.65; *p* = 0.68) and were below the IGSQ threshold of 23 commonly used to indicate certain GI distress. In contrast, the IGSQ composite score in FFI-Std was above the 23-cut-off indicating some GI discomfort (Figure 1).

### 3.3. Individual IGSQ Items and Additional GI-Tolerance Outcomes

Individual IGSQ item differences between the groups are shown in Table 2. FFI + LR and BFI had fewer hard stools than FFI-Std. Both FFI + LR and FFI-Std experienced more difficulty in passing stool than BFI. Compared to FFI-Std, FFI + LR experienced significantly fewer occasions of spitting-up, less spit-up on each occasion, less fussiness during spit-up and less total time crying in a day. FFI + LR was comparable to BFI with respect to these IGSQ items, while FFI-Std compared with BFI experienced more spit-up per occasion, more fussiness during spit-up and longer crying time. For occasions of spitting-up, FFI-Std were comparable with BFI. With respect to IGSQ items asking about soothing, overall fussiness or flatulence, arching back when spitting-up or crying directly after feeding, no significant differences were found among the groups, but some trends for less gassiness were observed in FFI + LR and BFI compared with FFI-Std (Table 2).

**Figure 2:**
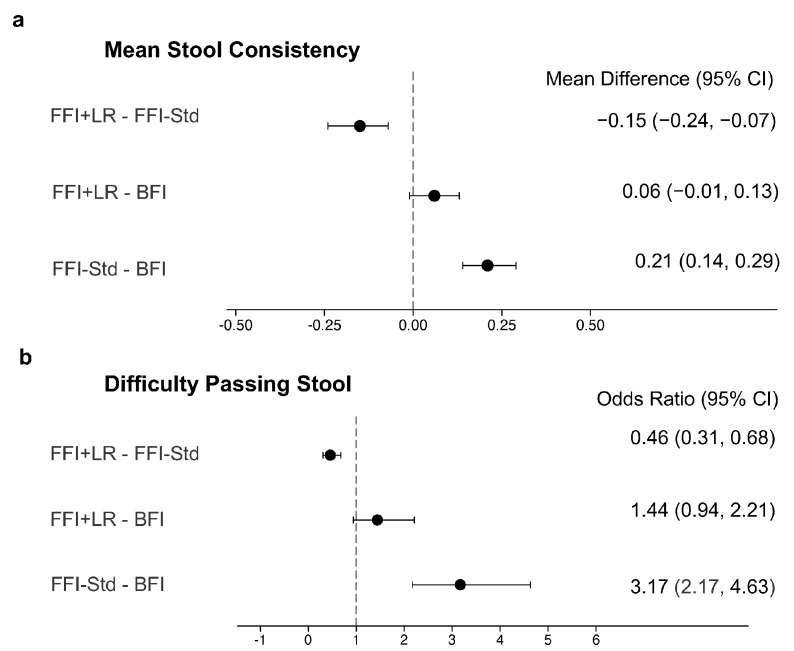
Comparisons of stool characteristics between the feeding groups, including mean differences for stool consistency (**a**) and odds ratios for difficulty passing stool (**b**). The vertical line shows the reference value for each measure. Stool consistency and difficulty in passing stool were measured using the Feeding Practice and Gut Comfort Questionnaire, which collected consistency for each stool and number of stools difficult to pass in the 24 h prior to the administration of the questionnaire. Stool consistency was measured using a 4-point scale (1 = Watery, 2 = Loose, 3 = Formed, 4 = Hard). Stool consistency was modeled using ANCOVA and difficulty passing stool was modeled using logistic regression. In addition to feeding group, models were further adjusted for study site (only for stool consistency), infant age, sex, delivery type, history of gastrointestinal disease in parents and mother’s education. BFI—Breastfed infants; FFI + LR—Infants fed formula with *L. reuteri*; FFI-Std—Infants fed standard formula without any probiotic or prebiotic. *n* = 760 in BFI; *n* = 501 in FFI-Std; *n* = 470 in FFI + LR.

For stooling pattern in the past 24 h based on the FPGCQ, mean stool consistency in FFI + LR was lower than in FFI-Std, indicating softer stools in FFI + LR compared to FFI-Std (Figure 2a). Mean stool consistency between FFI + LR and BFI was comparable with a trend for softer stools in BFI. FFI-Std had higher (i.e., harder) stool consistency than BFI. FFI + LR experienced significantly lower odds of difficulty in passing stool compared to FFI-Std (Figure 2b) and had comparable odds than BFI. FFI-Std had higher odds for difficulty in passing stool than BFI.

**Figure 3:**
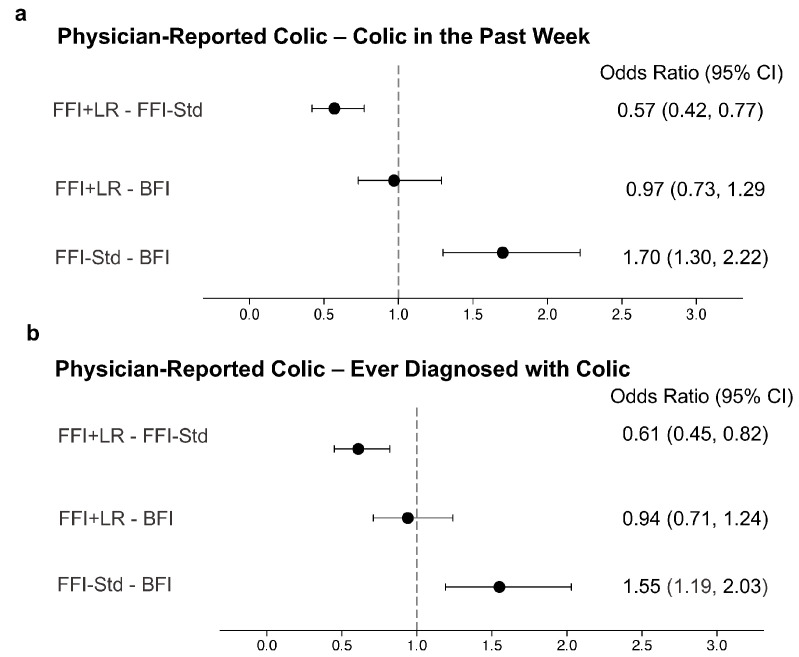
Odds ratios with 95% confidence interval comparing physician-reported colic between feeding groups. The vertical line shows the reference value for the odds ratios. Physician-reported colic was measured using the Feeding Practice and Gut Comfort Questionnaire (“Was your child ever diagnosed with colic?” and “Did the child have colic in the past week?”). Both outcomes were modeled using logistic regression and adjusted for study site, infant age, sex, delivery type, history of gastrointestinal disease in parents and mother’s education. BFI—Breastfed infants; FFI + LR—Infants fed formula with *L. reuteri*; FFI-Std—Infants fed standard formula without any probiotic or prebiotic. *n* = 760 in BFI; *n* = 501 in FFI-Std; *n* = 470 in FFI + LR. (**a**) Physician-reported colic-colic in the past week; (**b**)Physician-reported colic-ever diagnosed with colic.

Physician-confirmed colic in the past week prior to the study occurred in 23.6% of FFI + LR, 33.8% of FFI-Std and 23.6% of BFI, resulting in lower odds of colic in FFI + LR compared to FFI-Std (Figure 3a). FFI-Std had higher odds of colic in the past week compared to BFI while there was no difference between FFI + LR and BFI. Among the three groups, 28.1% of FFI + LR, 36.9% of FFI-Std and 28.4% of BFI were ever diagnosed with colic. Odds of having been ever diagnosed with colic were significantly lower in FFI + LR compared to FFI-Std (Figure 3b) and similar to BFI. Compared to BFI, FFI-Std had greater odds of ever being diagnosed with colic.

## 4. Discussion

A previously reported observational study found that GI tolerance was improved in infants who received formula containing any prebiotics or probiotics or a combination thereof compared with infants receiving formula that did not contain these ingredients [14]. The current secondary analysis of the same observational study provides interesting complementary data for one specific probiotic that has often been associated with GI tolerance. We found that formula containing *L. reuteri* DSM 17938 is associated with better GI tolerance, less difficulty in passing stool and reduced colic compared to formula without any probiotic or prebiotic. Further, infants receiving formula with *L. reuteri* had similar GI tolerance and prevalence of difficulty passing stools or colic as breastfed infants. Interestingly, the IGSQ composite score for breastfed infants and infants on *L. reuteri*-containing formula was below 23 indicating no GI distress, while for infants on standard formula without any probiotic or prebiotic, it was above 23, which is the threshold used to denote problematic tolerance with certain GI distress [15]. GI tolerance is an important factor for parents as signs of intolerance is a primary reason why they seek help from health care professionals and for infant formula switches [9,17]. Assessing GI tolerance, however, is challenging without an objective assessment tool. The use of a validated, standardized tool, such as the IGSQ in this study, provides a metric that is tangible and interpretable by clinicians and researchers and that allows for comparisons across studies. Indeed, the IGSQ composite score in our study for the *L. reuteri*-containing formula was similar to that from a real-world study for a formula containing *L. reuteri* and two human milk oligosaccharides reporting scores of 21.3 in mixed-fed infants and 22.7 in exclusively formula-fed infants at approximately 6 weeks of age [18]. Similarly, infants who switched to a formula containing *L. reuteri* and 2′fucosyllactose improved their IGSQ composite score from above 30 to 22.1 after receiving the *L. reuteri*-containing formula for 3 weeks [19]. Other studies conducted in the US and in China using the IGSQ to assess GI tolerance of formulas with optional ingredients reported lower composite scores [20,21]. Differences among studies might be explained by different effects of the various optional formula ingredients that were studied. As only 1 or 2 items in the IGSQ being answered differently could substantially affect the composite score, the sensitivity of the IGSQ must be considered when comparing across studies. Another reason for the differences observed among studies might be underlying baseline differences in diverse geographical regions.

Compared with formula without any probiotic or prebiotic, we found that *L. reuteri*-containing formula was associated with lower prevalence of colic and reduced crying time. These finding are consistent with a recent systematic review [22] that included 11 RCTs and 5 meta-analyses of probiotic use in relation to colic management. *L. reuteri* was the most commonly studied probiotic and the five meta-analyses examined oral administration of *L. reuteri* DSM 17938 or its mother strain *L. reuteri* ATCC 55730 in infants with colic. The meta-analyses showed strong evidence for the relief of colic symptoms in breastfed infants who received *L. reuteri* supplementation compared with placebo [23,24,25,26,27]. For formula-fed infants, for which a smaller number of studies was available than for breastfed infants, the evidence for reduced colic symptoms was moderate for probiotics [22] and data for *L. reuteri* was not conclusive. Our study provides effectiveness data that adds to the evidence base from RCTs and indicates a beneficial effect in formula-fed infants in terms of colic and crying when *L. reuteri* is part of a formula matrix. Additional clinical trials of formula-fed infants are still warranted to better understand the potential effects of *L. reuteri* on colic in this population.

*L. reuteri* has also been evaluated in prior trials in relation to spitting-up and results are consistent with the findings of our real-world study showing that infants receiving a *L. reuteri*-containing formula had fewer occasions of spitting-up than those receiving formula without any probiotic or prebiotic. In a randomized, double-blind trial of formula-fed infants with frequent regurgitation, the median number of episodes per day of spitting-up was reduced after 30 days in those receiving a *L. reuteri*-containing formula compared to those receiving control formula [28]. A randomized study of breastfed infants given *L. reuteri* or placebo also found a significant reduction in spitting-up after 28 days [29]. These effects might be explained by the influence *L. reuteri* has on the gastric emptying time [28].

In this study, we also observed differences in stooling patterns by feeding regimen. Stool consistency in infants receiving formula with *L. reuteri* pointed in the direction of breastfed infants (towards a softer consistency) and was indeed softer than in infants receiving formula without any probiotic or prebiotic. Additionally, difficulty in passing stools in infants who received formula with *L. reuteri* was similar to breastfed infants, but significantly lower compared with infants receiving formulas without any probiotic or prebiotic. It is hypothesized that probiotics may improve GI tract function and hence GI tolerance through several pathways. For example, *L. reuteri*, may help balance the gut microbiota by increasing beneficial bacteria and reducing pathogens [30] or may also strengthen the mucosal barrier [31]. These effects can influence gut motility [32] and hence stooling characteristics. Additionally, *L. reuteri* has been shown to reduce inflammatory markers such as calprotectin [33], possibly lowering the incidence of infectious diarrhea [34]. In exclusively breastfed colicky infants, *L. reuteri* supplementation reduced crying and/or fussing time [35,36,37], possibly by impacting gut microbiota composition, thus reducing abdominal gas and associated pain [34].

*L. reuteri* DSM 17938 has been examined in relation to multiple outcomes in clinical studies of infants [12]. However, most of the available data is for *L. reuteri* supplements in breastfed infants and real-world effectiveness data has only been published for infant formula combining *L. reuteri* and prebiotics [38] or for infant formula containing any pre- and/or probiotics [14] but not *L. reuteri* alone. Thus, the key strength of this study is its novelty providing the first large-scale real-world effectiveness data for one specific probiotic, *L. reuteri* DSM 17938, as part of a formula matrix covering a broad spectrum of endpoints including GI tolerance, stooling pattern and colic prevalence. Regarding colic, we collected information to examine both ever being diagnosed with colic and colic in the past week. The consistent findings for both outcomes—short- and long-term—strengthen the validity of the colic data. This study is additionally strengthened by its large sample size and the multi-country design which covered different lower or upper middle-income countries for which data on GI tolerance in formula-fed infants is limited. The study used a standardized, validated questionnaire to assess GI tolerance which allows for the comparison with other published literature that also utilized the IGSQ. One limitation of this study is the cross-sectional design; however, the IGSQ included a period of one week and in the FPGCQ, we retrospectively assessed colic prevalence over a longer period. Some statistically significant differences between the groups were observed in the baseline characteristics of the studied infants, hence ANCOVA models were adjusted notably for delivery type and mother’s education. Another limitation is that this was a secondary analysis of an observational study and thus the study design was not a priori powered for the specific outcomes examined herein.

## 5. Conclusions

In conclusion, in our real-world observational study, *L. reuteri*-containing formula was associated with improved overall digestive tolerance and behavioral patterns, softer stooling pattern and with reduced odds of physician-confirmed infantile colic. Our results add to the evidence base from RCTs examining *L. reuteri* DSM 17938, particularly for formula-fed infants. The similar conclusions drawn from our observational study conducted in a real-world setting and the existing body of RCTs are indicating that the observed effects of *L. reuteri* will translate into the broader population outside of a controlled clinical trial setting.

## Figures and Tables

**Table 1:** Demographic, anthropometric and parental characteristics of studied infants.

Characteristic	BFI	FFI + LR	FFI-Std
	*n* = 760	*n* = 470	*n* = 501
Age, days	82.1 (0.9)	80.2 (1.0)	82.5 (1.1)
Sex			
Female, %	44.6	48.5	43.7
Male, %	55.4	51.5	56.3
Delivery type *			
Caesarean, %	38.4	37.9	50.9
Vaginal, %	61.6	62.1	49.1
Gestational age at birth, weeks	38.7 (0.0)	38.5 (0.1)	38.5 (0.1)
Mother’s education *			
Low (primary school or lower), %	16.1	26.6	15.8
Medium (high school or professional school), %	42.0	46.8	45.5
High (college or higher), %	42.0	26.6	38.7
History of gastrointestinal disease in parents			
No, %	74.5	77.2	76.8
Yes, %	25.5	22.8	23.2
Birth weight *, g	3068 (18)	3001 (26)	2972 (21)
Birth length *, cm	49.3 (0.1)	49.2 (0.2)	48.6 (0.1)
Birth head circumference *, cm	33.5 (0.1)	33.6 (0.1)	33.9 (0.1)
Weight at visit **, g	5503 (35)	5137 (48)	5441 (42)
Length at visit **, cm	58.6 (0.2)	57.7 (0.2)	58.9 (0.2)
Head circumference at visit ***, cm	39.1 (0.1)	38.8 (0.1)	39.1 (0.1)

Data presented as mean (SE) or percentages, %. BFI—Breastfed infants; FFI + LR—Infants fed formula with *L. reuteri*; FFI-Std—Infants fed standard formula without any probiotic or prebiotic. * *p* < 0.01, ** *p* < 0.001, *** *p* < 0.05 for groupwise comparison using Chi-square test.

**Table 2:** Adjusted mean difference in individual IGSQ item scores between feeding groups ^1^.

IGSQ Domain	IGSQ Measure	Comparison	Difference of Adjusted Mean	95% CI	*p*-Value
Stool	1. Hard stools: “How many times did your baby pass a hard stool?”	FFI + LR–FFI-Std	−0.12	−0.21, −0.02	0.02
		FFI + LR–BFI	0.06	−0.02, 0.14	0.15
		FFI-Std–BFI	0.17	0.09, 0.26	<0.01
	2. Difficulty in passing stool: “How many times did your baby have difficulty when passing a bowel movement?”	FFI + LR–FFI-Std	−0.05	−0.18, 0.07	0.38
		FFI + LR–BFI	0.15	0.05, 0.26	<0.01
		FFI-Std–BFI	0.21	0.10, 0.31	<0.01
Spitting-up/Vomiting	3. Frequency of spit up: “How many times did milk come out of your baby’s mouth?”	FFI + LR–FFI-Std	−0.18	−0.34, −0.03	0.02
		FFI + LR–BFI	−0.09	−0.22, 0.05	0.22
		FFI-Std–BFI	0.10	−0.03, 0.23	0.15
	4. Volume of milk spit up: “How much milk usually came out each time?”	FFI + LR–FFI-Std	−0.20	−0.32, −0.08	<0.01
		FFI + LR–BFI	−0.08	−0.18, 0.03	0.14
		FFI-Std–BFI	0.12	0.02, 0.22	0.02
	5. Discomfort when spitting-up: “How often did your baby seem uncomfortable or fussy when milk came out of his or her mouth?”	FFI + LR–FFI-Std	−0.17	−0.29, −0.05	<0.01
		FFI + LR–BFI	−0.05	−0.15, 0.06	0.39
		FFI-Std–BFI	0.12	0.02, 0.23	0.02
	6. Frequency of arching back: “How many times did your baby arch his or her back as if in pain when milk came out of his or her mouth?”	FFI + LR–FFI-Std	−0.01	−0.11, 0.09	0.91
		FFI + LR–BFI	0.01	−0.08, 0.09	0.91
		FFI-Std–BFI	0.01	−0.07, 0.10	0.80
Crying	7. Total crying time: “How much total time did your baby usually cry in a day?”	FFI + LR–FFI-Std	−0.15	−0.28, −0.01	0.03
		FFI + LR–BFI	0.06	−0.06, 0.17	0.32
		FFI-Std–BFI	0.20	0.09, 0.32	<0.01
	8. Unable to soothe crying: “How many times were you unable to soothe your baby to stop his or her crying?”	FFI + LR–FFI-Std	−0.01	−0.16, 0.13	0.85
		FFI + LR–BFI	−0.10	−0.22, 0.03	0.12
		FFI-Std–BFI	−0.08	−0.20, 0.04	0.18
	9. Crying after feeding: “How many times did your baby cry during or right after a feeding because the milk bothered your baby?”	FFI + LR–FFI-Std	−0.08	−0.22, 0.06	0.26
		FFI + LR–BFI	−0.09	−0.21, 0.03	0.14
		FFI-Std–BFI	−0.01	−0.13, 0.11	0.87
Fussiness	10. Frequency of fussiness: “On how many days was your baby fussy?”	FFI + LR–FFI-Std	0.00	−0.15, 0.15	0.99
		FFI + LR–BFI	−0.01	−0.14, 0.12	0.89
		FFI-Std–BFI	−0.01	−0.13, 0.11	0.88
	11. Unable to soothe fussiness: “How many times were you unable to soothe your baby when he or she was fussy?”	FFI + LR–FFI-Std	−0.02	−0.15, 0.10	0.70
		FFI + LR–BFI	−0.03	−0.14, 0.08	0.62
		FFI-Std–BFI	0.00	−0.11, 0.11	0.95
Flatulence	12. Frequency of gassiness: “How many times in a usual day was your baby gassy?”	FFI + LR–FFI-Std	−0.16	−0.32, 0.00	0.05
		FFI + LR–BFI	−0.04	−0.18, 0.10	0.61
		FFI-Std–BFI	0.12	−0.01, 0.26	0.07
	13. Discomfort due to gas: “How often did gas seem to make your baby uncomfortable or fussy?”	FFI + LR–FFI-Std	−0.11	−0.25, 0.03	0.11
		FFI + LR–BFI	0.01	−0.11, 0.13	0.92
		FFI-Std–BFI	0.12	0.002, 0.24	0.05

BFI—Breastfed infants; CI—Confidence Interval; FFI + LR—Infants fed formula with *L. reuteri*; FFI-Std—Infants fed standard formula without any probiotic or prebiotic. IGSQ, Infant Gastrointestinal Symptom Questionnaire. *n* = 760 in BFI; *n* = 501 in FFI-Std; *n* = 470 in FFI + LR; *p*-values computed from ANCOVA models including feeding group as the independent variable and infant sex, age, study site, delivery type, history of gastrointestinal disease in parents and mother’s education as potential confounders. Higher scores indicate greater discomfort. Possible responses to questions 1, 2, 6, 8, 9, 11 were: 0 times, 1 time, 2–3 times, 4–6 times, 7 or more times in the week, or don’t know/no response. Possible responses for questions 3 and 12 were: 0 times, 1 time, 2–3 times, 4–6 times, 7 or more times in a usual day, or don’t know/no response. Possible responses to question 4 were: 5 mL, 15 mL, 30 mL, about half the feeding, more than half the feeding, or don’t know/no response. Possible responses to question 5 and 13 were: never, almost never, sometimes, almost always, always, or don’t know/no response. Possible responses to question 7 were: less than 10 min, 10–30 min, 30 min to 1 h, 1–2 h, 2 or more hours in a day, or don’t know/no response. Possible responses to question 10 were: 0, 1, 2, 3, 4, 5, 6, or 7 days. Reponses for individual questions were then scored according to predefined scoring guidelines.

## Data Availability

The datasets used and/or analyzed during the current study are available from the corresponding author on reasonable request.

## References

[1] Weaver L.T., Ewing G., Taylor L.C. (1988). The bowel habit of milk-fed infants. J. Pediatr. Gastroenterol. Nutr..

[2] Forsyth B.W., McCarthy P.L., Leventhal J.M. (1985). Problems of early infancy, formula changes, and mothers’ beliefs about their infants. J. Pediatr..

[3] Quinlan P.T., Lockton S., Irwin J., Lucas A.L. (1995). The Relationship between Stool Hardness and Stool Composition in Breast- and Formula-Fed Infants. J. Pediatr. Gastroenterol. Nutr..

[4] Ballard O., Morrow A.L. (2013). Human milk composition: Nutrients and bioactive factors. Pediatr. Clin. N. Am..

[5] Iacono G., Merolla R., D’amico D., Bonci E., Cavataio F., Di Prima L., Scalici C., Indinnimeo L., Averna M.R., Carroccio A. (2005). Gastrointestinal symptoms in infancy: A population-based prospective study. Dig. Liver Dis..

[6] Liu W., Xiao L.P., Li Y., Wang X.Q., Xu C.D. (2009). Epidemiology of mild gastrointestinal disorders among infants and young children in Shanghai area. Zhonghua Er Ke Za Zhi Chin. J. Pediatr..

[7] Iacovou M., Ralston R.A., Muir J., Walker K.Z., Truby H. (2012). Dietary management of infantile colic: A systematic review. Matern. Child Health J..

[8] Czinn S.J., Blanchard S. (2013). Gastroesophageal reflux disease in neonates and infants: When and how to treat. Paediatr. Drugs.

[9] Nevo N., Rubin L., Tamir A., Levine A., Shaoul R. (2007). Infant feeding patterns in the first 6 months: An assessment in full-term infants. J. Pediatr. Gastroenterol. Nutr..

[10] Alarcon P.A., Tressler R.L., Mulvaney A., Lam W., Comer G.M. (2002). Gastrointestinal tolerance of a new infant milk formula in healthy babies: An international study conducted in 17 countries. Nutrition.

[11] Kesavelu D., Sethi G., Bangale N., Anwar F., Rao S. (2018). Common gastrointestinal distress among infants: Role of optimal nutritional interventions. Clin. Epidemiol. Glob. Health.

[12] Urbanska M., Szajewska H. (2014). The efficacy of Lactobacillus reuteri DSM 17938 in infants and children: A review of the current evidence. Eur. J. Pediatr..

[13] Singal A.G., Higgins P.D., Waljee A.K. (2014). A primer on effectiveness and efficacy trials. Clin. Transl. Gastroenterol..

[14] Lavalle L., Sauvageot N., Cercamondi C.I., Egli D., Jankovic I., Vandenplas Y., Happy Tummy Consortium (2022). Infant feeding practice and gastrointestinal tolerance: A real-world, multi-country, cross-sectional observational study. BMC Pediatr..

[15] Riley A.W., Trabulsi J., Yao M., Bevans K.B., DeRusso P.A. (2015). Validation of a Parent Report Questionnaire: The Infant Gastrointestinal Symptom Questionnaire. Clin. Pediatr..

[16] Huysentruyt K., Koppen I., Benninga M., Cattaert T., Cheng J., De Geyter C., Faure C., Gottrand F., Hegar B., Hojsak I. (2019). The Brussels Infant and Toddler Stool Scale: A Study on Interobserver Reliability. J. Pediatr. Gastroenterol. Nutr..

[17] Polack F.P., Khan N., Maisels M.J. (1999). Changing partners: The dance of infant formula changes. Clin. Pediatr..

[18] Riechmann E.R., Villares J.M.M., Ortega F.D., Martínez A.C., Sirvent L.P., Santana L., Rivero J.C., Alshweki A., Cercamondi C., Dahbane S. (2020). Real-world study in infants fed with an infant formula with two human milk oligosaccharides. Nutr. Hosp..

[19] Czerkies L., Finn K.L., Kineman B.D., Reichert H., Cohen S.S., Carvalho R. (2019). Use of a partially hydrolyzed 100% whey-based infant formula with Lactobacillus reuteri in infants with caregiver-perceived intolerance. J. Pediatr. Health Nutr..

[20] Mao M., Zhang L., Ge J., Yan J., Northington R., Yao M., Nowacki J., Hays N.P. (2018). Infant Feeding Regimens and Gastrointestinal Tolerance: A Multicenter, Prospective, Observational Cohort Study in China. Glob. Pediatr. Health.

[21] Storm H.M., Shepard J., Czerkies L.M., Kineman B., Cohen S.S., Reichert H., Carvalho R. (2019). 2′-Fucosyllactose Is Well Tolerated in a 100% Whey, Partially Hydrolyzed Infant Formula With Bifidobacterium lactis: A Randomized Controlled Trial. Glob. Pediatr. Health.

[22] Simonson J., Haglund K., Weber E., Fial A., Hanson L. (2021). Probiotics for the Management of Infantile Colic: A Systematic Review. MCN Am. J. Matern. Child Nurs..

[23] Dryl R., Szajewska H. (2018). Probiotics for management of infantile colic: A systematic review of randomized controlled trials. Arch. Med. Sci..

[24] Gutierrez-Castrellon P., Indrio F., Bolio-Galvis A., Jimenez-Gutierrez C., Jimenez-Escobar I., Lopez-Velazquez G. (2017). Efficacy of Lactobacillus reuteri DSM 17938 for infantile colic: Systematic review with network meta-analysis. Medicine.

[25] Schreck Bird A., Gregory P.J., Jalloh M.A., Risoldi Cochrane Z., Hein D.J. (2017). Probiotics for the Treatment of Infantile Colic: A Systematic Review. J. Pharm. Pract..

[26] Sung V., D’Amico F., Cabana M.D., Chau K., Koren G., Savino F., Szajewska H., Deshpande G., Dupont C., Indrio F. (2018). Lactobacillus reuteri to Treat Infant Colic: A Meta-analysis. Pediatrics.

[27] Xu M., Wang J., Wang N., Sun F., Wang L., Liu X.H. (2015). The Efficacy and Safety of the Probiotic Bacterium Lactobacillus reuteri DSM 17938 for Infantile Colic: A Meta-Analysis of Randomized Controlled Trials. PLoS ONE.

[28] Indrio F., Riezzo G., Raimondi F., Bisceglia M., Filannino A., Cavallo L., Francavilla R. (2011). Lactobacillus reuteri accelerates gastric emptying and improves regurgitation in infants. Eur. J. Clin. Investig..

[29] Garofoli F., Civardi E., Indrio F., Mazzucchelli I., Angelini M., Tinelli C., Stronati M. (2014). The early administration of Lactobacillus reuteri DSM 17938 controls regurgitation episodes in full-term breastfed infants. Int. J. Food Sci. Nutr..

[30] Savino F., Fornasero S., Ceratto S., De Marco A., Mandras N., Roana J., Tullio V., Amisano G. (2015). Probiotics and gut health in infants: A preliminary case–control observational study about early treatment with Lactobacillus reuteri DSM 17938. Clin. Chim. Acta.

[31] Rosenfeldt V., Benfeldt E., Valerius N.H., Paerregaard A., Michaelsen K.F. (2004). Effect of probiotics on gastrointestinal symptoms and small intestinal permeability in children with atopic dermatitis. J. Pediatr..

[32] Wu R.Y., Pasyk M., Wang B., Forsythe P., Bienenstock J., Mao Y.K., Sharma P., Stanisz A.M., Kunze W.A. (2013). Spatiotemporal maps reveal regional differences in the effects on gut motility for Lactobacillus reuteri and rhamnosus strains. Neurogastroenterol. Motil..

[33] Savino F., Garro M., Montanari P., Galliano I., Bergallo M. (2018). Crying Time and RORgamma/FOXP3 Expression in Lactobacillus reuteri DSM17938-Treated Infants with Colic: A Randomized Trial. J. Pediatr..

[34] Alam M., Islam M., Ziaul M., Tayab M., Alam K., Sahid H., Kamrul M., Mahmood S., Haque A. (2022). Role of Probiotic Lactobacillus reuteri in Improving Gut Health and Immunity in Infants and Toddlers: A Review. Int. J. Nutr. Sci..

[35] Chau K., Lau E., Greenberg S., Jacobson S., Yazdani-Brojeni P., Verma N., Koren G. (2015). Probiotics for infantile colic: A randomized, double-blind, placebo-controlled trial investigating Lactobacillus reuteri DSM 17938. J. Pediatr..

[36] Mi G.L., Zhao L., Qiao D.D., Kang W.Q., Tang M.Q., Xu J.K. (2015). Effectiveness of Lactobacillus reuteri in infantile colic and colicky induced maternal depression: A prospective single blind randomized trial. Antonie Van Leeuwenhoek.

[37] Savino F., Cordisco L., Tarasco V., Palumeri E., Calabrese R., Oggero R., Roos S., Matteuzzi D. (2010). Lactobacillus reuteri DSM 17938 in infantile colic: A randomized, double-blind, placebo-controlled trial. Pediatrics.

[38] Vandenplas Y., Gerlier L., Caekelbergh K., Possner M., Nan-Study-Group (2021). An Observational Real-Life Study with a New Infant Formula in Infants with Functional Gastro-Intestinal Disorders. Nutrients.

